# Harnessing the power of natural killer cells in Lymphoma immunotherapy: Overcoming challenges and enhancing treatment options

**DOI:** 10.1016/j.htct.2026.106352

**Published:** 2026-05-04

**Authors:** Elham Roshandel, Maryam Vahdat Lasemi, Maryam Mehravar, Maryam Salimi, Mohammad Majidi, Fatemeh Mohammadali, Hamide Rahmani Seraji, Abbas Hajifathali

**Affiliations:** a Hematopoietic Stem Cell Research Center, Shahid Beheshti University of Medical Sciences, Tehran, Iran; b Department of Tissue Engineering & Regenerative Medicine, Faculty of Advanced Technologies in Medicine, Iran University of Medical Sciences, Tehran, Iran; c Blood Transfusion Research Center, High Institute For Research and Education in Transfusion Medicine, Tehran, Iran; d Department of Hematology and Oncology, Taleghani Hospital, Shahid Beheshti University of Medical Sciences, Tehran, Iran

**Keywords:** Immunotherapy, Lymphoma treatment, NK cell therapy, Tumor microenvironment

## Abstract

Immunotherapy, particularly natural killer cell therapy, is garnering attention for treating lymphoma, due to the unique properties of natural killer cells, most importantly rapid and potent antitumor responses without prior sensitization. However, the tumor microenvironment and immune escape mechanisms can impair natural killer cell function in lymphoma.

To address these challenges, researchers are exploring therapeutic interventions to restore or enhance their activity. Of the various approaches of cell therapy, such as allogeneic- or autologous-natural killer cell infusions, chimeric antigen receptor natural killer cells, and combination therapies, allogeneic natural killer cell transplantation has shown promise in specific subtypes of lymphoma, offering a reduced risk of graft-versus-host disease, improved response rates, prolonged remissions, and increased overall survival. Combining natural killer cell therapy with standard treatments such as chemotherapy or immune checkpoint inhibitors holds potential for synergistic effects. Nevertheless, addressing challenges such as cell persistence, the immunosuppressive tumor microenvironment, and optimal delivery methods is crucial to improve efficacy.

Further investigations are required to gain a better understanding of natural killer cell-mediated responses, refine genetic engineering approaches, identify predictive biomarkers, and optimize combination strategies. Continued research and clinical trials will play a vital role in optimizing cell therapy and expanding treatment options for lymphoma patients.

## Introduction

Lymphoma is a general category of hematological malignancies that arise from aberrant lymphocytes, which are part of the immune system. Lymphomas are typically divided into Hodgkin’s lymphoma (HL) and non-Hodgkin lymphoma (NHL), the latter being much more common than the former. Among the subtypes of NHL, the most common are B-cell lymphomas, which include diffuse large B-cell lymphoma (DLBCL), follicular lymphoma, and mantle cell lymphoma. In contrast, T-cell lymphomas and HL are less common but have important clinical subtypes with unique pathologic and clinical features [1]. Despite advances in chemotherapy and targeted therapy regimens, the outlook for the vast majority of patients with relapsed or refractory B-cell lymphoma remains unfavorable. Standard first-line treatment regimens, including R-CHOP (rituximab, cyclophosphamide, doxorubicin, vincristine, and prednisone) have dramatically increased survival rates in B-cell lymphomas, particularly diffuse large B-cell lymphoma (DLBCL). However, these regimens have limited long-term benefits in patients with high-risk or advanced disease. In addition, autologous stem cell transplantation, although beneficial for some, is not an option in all cases. Similarly, resistance to treatment and relapse are often seen in other types of lymphoma, such as some T-cell lymphomas and HLs [2]. These limitations highlight the need for more robust and individualized treatment approaches across the spectrum of lymphatic malignancies.

Cell-based immunotherapy has revolutionized cancer treatment by leveraging immune system cells to identify and eradicate tumor cells. Among the diverse cell-based immunotherapeutic strategies, the utilization of natural killer (NK) cells has emerged as a promising therapeutic approach in lymphoma. As innate immune effectors, NK cells possess distinctive properties that render them attractive candidates for immunotherapy, notably their capacity to recognize and eliminate tumor cells without prior sensitization. NK cells play a critical role in the immune surveillance against tumor development and viral infections. They possess a delicate balance of activating and inhibitory receptors that allows them to distinguish between healthy cells and those that exhibit altered self-characteristics, such as infected or malignant cells [3].

However, in the context of lymphoma, aberrations within the tumor microenvironment can suppress NK cell activity, compromising their ability to effectively target and eliminate tumor cells. Thus, strategies to enhance NK cell function and overcome immunosuppressive mechanisms are being explored to unleash the full potential of these cells in lymphoma treatment. These strategies include targeting immunosuppressive factors, modulating cytokine levels, and overcoming NK cell exhaustion. By understanding the complex interplay between NK cells and the lymphoma microenvironment, novel immunotherapeutic strategies can be developed to improve anti-tumor responses and enhance NK cell-mediated immunity in lymphoma.

## Overview of natural killer cell immunotherapy in lymphoma

### *Natural killer cell phenotypic changes during lymphomagenesis*

NK cells are a type of CD16^+^ CD56^+^ cytotoxic lymphocyte that belong to the innate immune system. These cells have the ability to induce apoptosis even in the absence of antibodies and major histocompatibility complexes. NK cells recognize tumor antigens through killer-cell immunoglobulin-like receptors (KIRs), which have both inhibitory or activating functions depending on the intracytoplasmic region of the receptor. The presence and behavior of NK cells can be altered in the site where lymphoma is initiated. Lymphomas develop complex interactions with the immune system, including alterations in the microenvironment where lymphomas develop, that impact NK cell function and behavior. Multiple modifications occur in the lymphoma microenvironment, including changes in immunoregulatory cells, the cytokine milieu, stromal cells, expression of activating and inhibitory receptors, and functional NK cells [4]. Variations in NK cells within the lymphoma microenvironment differ based on the particular subtype of lymphoma. Classic HL (cHL) is characterized by a potent immunosuppressive tumor microenvironment (TME) that inhibits NK cells. The immunosuppressive nature of the TME in cHL specifically inhibits the proliferation and activity of NK cells, thereby contributing to the tumor immune-escape mechanisms. This deficiency of NK cells begins at the tumor site and progresses systemically in patients with advanced disease or adverse prognostic factors. Several facets of cHL account for this effect on NK cells. Locally, malignant Reed-Sternberg cells and cells from the TME express ligands for inhibitory receptors on NK cells, including Human Leukocyte Antigen (HLA)-E, HLA-G, and programmed death-ligand 1. The secretion of chemokines and cytokines, including soluble interleukin (IL)-2 receptor (sCD25), transforming growth factor-β, IL-10, CXCL9, and CXCL10, mediates the systemic immunosuppression [5]. However, generally in the lymphoma spectrum in terms of immunoregulatory cells, the lymphoma microenvironment often contains regulatory T cells (Tregs) and myeloid-derived suppressor cells (MDSCs). These cells suppress NK cell activity and impair their antitumor functions, leading to immune evasion by lymphoma cells [6]. Besides immunoregulatory cells, stromal cells in the lymphoma microenvironment, such as cancer-associated fibroblasts (CAFs), secrete factors that suppress NK cell function. They may also contribute to the formation of a physical barrier, limiting NK cell infiltration into the tumor site [7]. In addition to cells, the presence of certain cytokines within the lymphoma microenvironment influence NK cell behavior. Elevated levels of transforming growth factor-beta (TGF-β) and IL-10 inhibit NK cell function, impairing their cytotoxicity and cytokine production. The well-characterized inhibitory effect of TGF-β on the effector functions and cytotoxic activity of NK cells has been demonstrated. It has been shown that the IL-10 signaling pathway can be promoted to inhibit the TGF-β-induced suppression of NK cell cytotoxic function [8]. NK cells also express an array of activating and inhibitory receptors that regulate their activity. In lymphomas, the expression of these receptors on NK cells can be altered, affecting their ability to recognize and kill tumor cells. For example, the downregulation of NKG2D receptor ligands on lymphoma cells impair NK cell activation [9]. Notably, prolonged exposure to the lymphoma microenvironment leads to NK cell exhaustion or dysfunction. This exhaustion is characterized by reduced cytotoxicity, impaired cytokine production, and altered receptor expression. Exhausted NK cells exhibit decreased responsiveness to stimulation and diminished tumor-killing capacity [10]. Understanding the complex interplay between NK cells and the lymphoma microenvironment is crucial for developing novel immunotherapeutic strategies to enhance NK cell function and improve anti-tumor responses.

## How natural killer cells are suppressed in lymphoma

Despite the importance of NK cells in the immune response against cancer cells, lymphomas employ various mechanisms to evade or suppress NK cell activity. The most common suppressive factors are tumor-derived soluble factors, which are released by lymphoma cells and have immunosuppressive effects on NK cells. These factors include cytokines, chemokines, and growth factors, such as TGF-β and IL-10, which inhibit the activity of NK cells and impair their cytotoxic functions [11]. The interaction between inhibitory receptors expressed on NK cells and their corresponding ligands on lymphoma cells represents another mechanism that suppresses NK cell activity. Inhibitory receptors recognize specific ligands on target cells, and lymphoma cells can upregulate the expression of ligands for these inhibitory receptors, such as Major Histocompatibility Complex class I molecules. These ligands engage with inhibitory receptors like KIRs and NKG2A, transmitting inhibitory signals to the NK cells and dampening their cytotoxicity against lymphoma cells [12]. In addition, immunoregulatory cells, such as Tregs and MDSCs, that often exist in the lymphoma microenvironment also suppress NK cell function. These cells produce immunosuppressive molecules and inhibit NK cell activity, preventing effective anti-tumor responses [13]. Furthermore, extracellular matrix components and stromal cells in the lymphoma microenvironment, such as cancer-associated fibroblasts (CAFs), secrete factors and remodel the extracellular matrix, creating a physical barrier that hampers NK cell infiltration into the tumor site. This limited access to malignant lymphocytes imposes spatial constraints that impede NK cell recognition and subsequent mediated cytotoxicity [14]. Metabolic reprogramming derived from lymphoma cells also leads to alterations in the tumor microenvironment that affect NK cell function. For example, increased consumption of nutrients and competition for resources within the microenvironment impair the NK cell metabolism and compromise their effector functions [15]. Immune checkpoint molecules, such as programmed cell death protein 1 (PD-1) and cytotoxic T-lymphocyte-associated protein 4 (CTLA-4), expressed in NK cells are upregulated in the lymphoma microenvironment [16]. Engagement of these checkpoints by their ligands on lymphoma cells leads to NK cell exhaustion and functional suppression. These immune checkpoint molecules are negative regulators of T-cell and NK cell function that limit the amplitude of T cell and NK cell activation. The blockade of immune checkpoints, such as PD-1 and CTLA-4, has shown promising results in enhancing antitumor immunity and producing durable clinical responses [17]. Moreover, genetic and epigenetic changes within lymphoma cells contribute to NK cell suppression. These alterations affect the expression of molecules involved in NK cell recognition and activation, impairing their ability to effectively target and eliminate lymphoma cells. It has been observed that promoter methylation/hypermethylation of tumor suppressor genes, miRNA expression or methylation level, and Long noncoding RNA (LncRNA) expression were associated with lymphoma disease progression [18].

## How medications affect the activation of natural killer cells

The treatment of lymphoma relies heavily on medication, which varies depending on the type and stage of lymphoma, as well as individual patient factors. Common types of medication used in lymphoma treatment include chemotherapy, monoclonal antibodies, immunomodulatory drugs, and targeted therapies [19].

### *Chemotherapy*

Some chemotherapy agents have suppressive effects on NK cell function. Investigations have been conducted on the effects of the chemotherapy agents commonly used in lymphoma treatment on NK cell proliferation and cytotoxicity. The findings indicate that NK cell activation is modulated by chemotherapy-induced DNA damage and DNA damage response pathways, leading to alterations in the NK cell phenotype and impaired cytotoxicity [20]. Research has been carried out on the impact of various chemotherapy drugs, such as cyclophosphamide and methotrexate, on the ability of NK cells to destroy cancer cells. These investigations have focused on the role of a protein called NKG2D ligand in mediating this process. The findings have shown that specific chemotherapy agents cause a decrease in the expression of NKG2D ligands on lymphoma cells. As a result, NK cells have a reduced ability to recognize and eliminate these cancer cells [21]. Moreover, when both dendritic cells (DCs) and NK cells were treated with chemotherapy such as ifosfamide, DCs were able to overcome the negative effect of ifosfamide on NK cytotoxic function. It has been reported that Ifosfamide treatment of DCs resulted in a reduction in indirect DC stimulation of NK cell proliferation via T cells and T cell-derived IL-2. While the negative impact on NK cytotoxic function can be overcome by DC, the restoration of NK cell interferon-gamma (IFN-γ) production is not as effective [22].

### *Monoclonal antibodies*

While chemotherapy may have suppressive effects on NK cell function, monoclonal antibody therapies enhance NK cell-mediated cytotoxicity. Rituximab, an anti-CD20 chimeric monoclonal antibody, targets the CD20 protein found on the surface of B cells, including malignant B cells in some lymphomas. Studies suggest that rituximab enhances NK cell-mediated cytotoxicity through the CD16 signaling pathway, which is involved in antibody-dependent cellular cytotoxicity (ADCC). Rituximab-mediated binding to CD20 on lymphoma cells triggers NK cell activation and ADCC, ultimately resulting in the targeted elimination of malignant cells. The results of a meta-analysis confirmed that NK cell-mediated cytotoxicity against lymphoma cells through ADCC is significantly enhanced by rituximab treatment thereby contributing to improved clinical outcomes [23].

### *Immunomodulatory drugs*

Between 2012 and 2014, research was focused on immunomodulatory medications in lymphoma, including lenalidomide. Lenalidomide has immunomodulatory effects, including the activation and enhancement of NK cell function, stimulating NK cell activity and cytokine production, potentially contributing to antitumor immune responses. Lenalidomide treatment increases CD20 expression on lymphoma cells, leading to enhanced NK cell-mediated lysis of tumor cells. It also enhances NK cell activity, including increased cytotoxicity and IFN-γ production and modulates cytokine production by ovarian cancer cells. These studies provide evidence that lenalidomide can activate and enhance NK cell function, contributing to antitumor immune responses in lymphoma treatment [23]. The immunomodulatory effects of lenalidomide and its potential contribution to antitumor immune responses in lymphoma treatment are supported by these findings. Furthermore, recent studies suggest that the combination of lenalidomide and rituximab may have complementary mechanisms of action as this unique combination is characterized by immune enhancement, not immunosuppression. This benefit was demonstrated in the Phase III AUGMENT trial, which evaluated rituximab with or without lenalidomide in relapsed rituximab-sensitive follicular lymphoma, and found a significant progression-free survival benefit with the combination. Results demonstrated that lenalidomide has synergy with anti-CD20 antibodies such as obinutuzumab and rituximab, via reconstitution of a functional immune synapse, improved NK cell function, and greater antibody-dependent cellular cytotoxicity [24]. In animal models and *in vitro* studies, the effect of rituximab in combination with lenalidomide on ADCC and direct apoptosis was assessed in B-cell lymphoma. The results showed that the lenalidomide and rituximab combination therapy enhanced ADCC and direct apoptosis in B-cell lymphoma [25]. Multiple phase II studies showed that lenalidomide and rituximab therapy achieves clinical synergy in treatment naïve, relapsed and refractory settings [26].

### *Targeted therapy*

Unlike chemotherapy, which can affect both cancerous and healthy cells, targeted therapies are more selective in their action and can minimize damage to healthy cells. They specifically target molecules or pathways involved in the immunosuppressive microenvironment and be used alone or in combination with other treatments, such as chemotherapy or immunotherapy. Various targeted therapies, such as kinase inhibitors, are now used in the treatment of specific lymphoma subtypes including venetoclax, duvelisib, copanlisib, polatuzumab vedotin, and ibrutinib. Venetoclax, duvelisib, and copanlisib inhibit some factors that are involved in the growth and survival of cancer cells [27]. Similarly, ibrutinib disrupts B-cell receptor (BCR) signaling by inhibiting Bruton’s tyrosine kinase (BTK), thereby impairing the essential growth and survival mechanisms of malignant B cells. By inhibiting this signaling pathway, ibrutinib helps to slow down or stop the growth of lymphoma cells. Brentuximab vedotin and polatuzumab vedotin target proteins found on the surface of some lymphoma cells. These targeted therapies may indirectly facilitate NK cell activation by disrupting tumor-mediated immune evasion pathways and reducing the overall suppressive burden within the microenvironment [28].

Recent studies have investigated the potential of various inhibitors to enhance NK cell-mediated cytotoxicity against lymphoma cells. One study found that Bruton's tyrosine kinase (BTK) inhibitors, such as ibrutinib, can enhance NK cell-mediated cytotoxicity by targeting signaling pathways involved in immune evasion and tumor cell survival. BTK, an essential component of the B-cell receptor pathway, has emerged as a novel target in the treatment of B-cell malignancies [29].

In addition, selinexor, a selective inhibitor of the nuclear export protein exportin-1 (XPO1), enhances NK cell activation against malignant B cells via downregulation of HLA-E. The NKG2A:HLA-E axis is a novel immune checkpoint target; it has been found that selinexor sensitizes lymphoma cells to NK cell-mediated killing via disruption of this interaction. The study also provides preliminary evidence suggesting an association between NK cell activity and clinical response to selinexor. These data indicate that NK cells may contribute to the therapeutic efficacy of selinexor, potentially providing a rationale for its synergistic use with NK cell-targeted therapies in lymphoma treatment [30].

## Natural killer cell role as a therapy for lymphoma

NK cells are crucial in host immunity against cancer, but cancers develop mechanisms to escape NK cell attack or induce defective NK cells. NK cell-based cancer immunotherapy aims to overcome NK cell paralysis using several approaches. These approaches include expanded allogeneic NK cells, stable allogeneic NK cell lines, and genetic modification of fresh NK cells or NK cell lines. Therapeutic NK cells can be derived from various sources, including peripheral or cord blood cells, stem cells, or even induced pluripotent stem cells (iPSCs) [31]. A variety of stimulators can be used for large-scale production in laboratories or good manufacturing practice facilities, including soluble growth factors, immobilized molecules or antibodies, and other cellular activators. In the first approach, expanded allogeneic NK cells are not inhibited by self-histocompatibility antigens like autologous NK cells, making them suitable for adoptive cellular immunotherapy. In the second approach, stable allogeneic NK cell lines are more practical for quality control and large-scale production. Thirdly, genetic modification of fresh NK cells or NK cell lines to highly express cytokines, Fc receptors, and/or chimeric tumor-antigen receptors is another approach [31]. In the context of lymphoma, the objective of NK cell therapy revolves around exploiting the inherent anti-tumor attributes possessed by these cells, with the specific aim of selectively targeting and eradicating lymphoma cells. The implementation of this therapy entails the introduction of *ex vivo* expanded and activated autologous (derived from the patient themselves) or allogeneic (derived from a donor) NK cells into the patient's system [32], as illustrated in Table 1. The ultimate goal lies in bolstering the immune response against lymphoma cells, thereby promoting regression of the tumor. Clinical trials investigating allogeneic NK cell therapies in patients with relapsed or refractory hematologic malignancies, including those with B-cell lymphoma, have supported the significant anti-tumor activity of these cells. These therapies may provide a superior safety profile, as they are associated with a lower incidence of cytokine release syndrome (CRS) and neurologic toxicity compared to T-cell therapies. Nevertheless, the use of allogeneic NK cell therapies is hampered by several limitations, such as the scarcity of appropriate donors, the relatively short *in vivo* persistence, and the manufacturing constraints that hinder the consistent administration of multiple doses [33].

**Table 1:** Diverse therapeutic strategies utilizing natural killer cell therapy for lymphoma.

Therapy Approach	Description	Advantages	Challenges
Allogeneic NK Cell Transplantation	Infusion of NK cells from a healthy donor	Reduced risk of graft-versus-host disease (GvHD)	Donor availability and compatibility
Autologous NK Cell Infusion	Infusion of patient's own NK cells	Lower risk of immune rejection	Limited NK cell numbers and quality
CAR-NK Cell Therapy	Genetic engineering of NK cells to express CARs	Enhanced specificity and potency	Optimization of CAR design and manufacturing
Combination Therapies	Co-administration of NK cell therapy with other treatments	Synergistic effects for improved outcomes	Identifying optimal combination strategies
Cytokine Stimulation	Administration of cytokines (*e.g.*, IL-2, IL-15)	Promotes NK cell proliferation and cytotoxicity	Potential systemic side effects
Antibody Stimulation	Administration of antibodies (*e.g.*, anti-CD20)	Enhances NK cell-mediated antibody-dependent cytotoxicity (ADCC)	Resistance or downregulation of target antigens
Gene Editing Techniques	CRISPR/Cas9-mediated gene editing in NK cells	Improved persistence and resistance to immunosuppression	Off-target effects and safety concerns

NK cell therapy for lymphoma can be administered either as a standalone treatment or in conjunction with other therapeutic modalities such as chemotherapy, radiation therapy, or targeted therapies. The intention behind combination approaches is to amplify the effectiveness of standard treatments while concurrently enhancing the immune response against lymphoma [34]. Table 2 outlines selected clinical studies using allogeneic NK cells in lymphoma, while Table 3 provides preclinical data supporting their potential efficacy.

**Table 2:** Clinical trials of allogeneic natural killer cell therapy in lymphoma.

Clinical Trial ID	Phase	Sponsor	Product Name	CAR Target	NK Cell Source	Indication	Outcome Summary
NCT04074746	I/II	MD Anderson Cancer Center	AFM13-NK	CD30	Cord blood	Relapsed/refractory Hodgkin’s and non-Hodgkin lymphomas	ORR: 89 %; CR: 53 %; No major adverse events
NCT05883449	I/II	MD Anderson Cancer Center	Precomplexed AFM13 + CIML NK Cells	CD30	Cord blood	Relapsed/refractory CD30^+^ lymphomas	ORR: 93 %; CR: 66 %; Favorable safety profile
NCT04245722	I	Fate Therapeutics	FT596	CD19 + IL-15	iPSC-derived	Non-Hodgkin Lymphoma	Early-phase study; outcomes pending
NCT05020015	II	Takeda	TAK-007	CD19	Cord blood	Relapsed/refractory LBCL and iNHL	ORR: 78 % in iNHL; CR: 56 %; No severe CRS or ICANS

CAR: chimeric antigen receptor; CD: cluster of differentiation; CR: complete response; ORR: overall response rate; NK: natural killer; CIML: cytokine-induced memory-like; iPSC: induced pluripotent stem cell; LBCL: large B-cell lymphoma; iNHL: indolent non-Hodgkin lymphoma; CRS: cytokine release syndrome; ICANS: immune effector cell-associated neurotoxicity syndrome.

**Table 3:** Preclinical studies of allogeneic natural killer cells in lymphoma.

NK Cell Source	Engineering	Disease Model	Strategy	Key Findings
Cord Blood	CIML NK Cells + AFM13	CD30+ Lymphoma (Mouse Model)	Combination Therapy	Enhanced cytotoxicity; maintained efficacy in suppressive tumor microenvironment
Peripheral Blood	IL-12/15/18 Activated NK Cells	Lymphoma/Melanoma (Xenograft)	Cytokine-Induced Memory-Like NK Cells	Increased IFN-γ production; improved persistence and antitumor activity

NK: Natural killer cell; CIML: Cytokine-induced memory-like; CAR: Chimeric antigen receptor; TME: Tumor microenvironment; IFN-γ: Interferon gamma.

## Chimeric antigen receptor-natural killer cell therapy in lymphoma

Chimeric Antigen Receptors (CARs) are genetically engineered to recognize and bind to antigens on the cell surface. Receptors allow cells to recognize specific targets, for example, tumor cells, and trigger potent cytotoxic activity against them. CARs are usually designed to bind to particular antigens on the surface of cancer cells and therefore provide targeted therapies against tumors. While the earliest cancer treatments were mainly based on CAR-T cell therapy, recent studies have also proposed CAR-NK cell therapy (CAR-engineered NK cells) as a promising candidate for tumor treatment [35].

These treatments make use of NK cells, which already have the innate ability to recognize and eradicate cancer cells. By using CAR technology, the cells are engineered to recognize and eliminate specific tumor antigens. These cells, due to their unique features, *i.e.*, not requiring HLA matching and possessing allogeneic potential, can effectively target cancer cells. Furthermore, the ability of CAR-NK cells to exert potent antitumor effects without inducing severe toxicities (specifically CRS, neurotoxicity, or graft-versus-host disease [GvHD]) establishes this modality as a safe and efficacious alternative to traditional cellular therapies [36].

The efficacy of CAR-NK cells in treating lymphoma and other hematologic malignancies is being investigated in several studies. A pivotal study by Liu et al., published in The New England Journal of Medicine, demonstrated that cord blood-derived CAR-NK cells targeting CD19 induced high response rates without CRS, neurotoxicity, or GvHD; this represents a significant milestone in NK cell therapy development (Clinical Trial: NCT03056339) [37].

Building upon this foundational work, Rezvani et al. reported the successful application of off-the-shelf allogeneic CAR-NK cells with sustained efficacy and favorable safety profiles in patients with relapsed or refractory lymphoid malignancies, including lymphoma. These findings further highlighted the feasibility and clinical relevance of CAR-NK cells as a scalable, low-toxicity alternative to CAR-T cell therapies [38]. One of the most promising clinical trials was conducted by Fate Therapeutics, in which iPSC-derived CAR-NK cells (FT596) were administered to treat relapsed and refractory B-cell lymphomas. FT596 was designed to target three antigens, CD19, CD16, and IL-15 in order to increase anti-tumor activity and persistence. The trial enrolled 86 patients across nine US research sites. The outcomes revealed that 50 % of the patients who received a high dose of FT596 (900 million cells) and 33 % of the patients receiving lower doses achieved a complete response. Side effects were low, primarily including mild CRS that resolved without intensive care. Notably, no cases of immune effector cell-associated neurotoxicity syndrome (ICANS) or GvHD were observed (Clinical Trial: NCT04245722) [39]. Further advancements were made by Nkarta Therapeutics, who conducted trials using their allogeneic CAR-NK product, NKX019, targeting CD19 antigens. This trial demonstrated an impressive overall response rate (ORR) of approximately 80 % and a complete response (CR) of 70 %. Most importantly, no CRS or neurotoxicity occurred, confirming both the efficacy and safety (Clinical Trial: NCT05020678) [40]. Similarly, the TAK-007 (ARC-TCR) trial employed umbilical cord blood-derived CAR-NK cells for the treatment of indolent NHL (iNHL) and large B-cell lymphoma (LBCL). Preliminary results indicated an ORR of 78 % in iNHL and 50 % in LBCL, again without major side effects such as CRS and neurotoxicity (Clinical Trial: NCT05020015) [41]. A more recent approach by Allogene Therapeutics involved the use of allogeneic CAR-NK cells (ALLO-316) specifically engineered to target CD19 antigens in refractory B-cell lymphomas. This study reported a 60 % ORR, with no high-grade toxicities like CRS or neurotoxicity, positioning ALLO-316 as a promising novel therapy in the management of difficult-to-treat lymphomas (Clinical Trial: NCT04263594) [42]. Key clinical trials evaluating CAR-NK cell therapy in B-cell lymphoma are summarized in Table 4.

**Table 4:** Clinical trials of chimeric antigen receptor-natural killer cell therapy in lymphoma.

Clinical Trial ID	Phase	Sponsor	Product Name	CAR Target	NK Cell Source	Indication	Outcome Summary
NCT05020015	II	Takeda	TAK-007	CD19	Cord Blood	Relapsed/refractory LBCL and iNHL	ORR: 78 % (iNHL); CR: 56 %; No CRS or ICANS
NCT04245722	I	Fate Therapeutics	FT596	CD19 + IL-15	iPSC-derived	Relapsed/refractory B-cell NHL	CR in 50 % (high dose); safe profile
NCT05020678	I	Nkarta Inc.	NKX019	CD19	Peripheral Blood	Relapsed/refractory B-cell lymphoma	ORR: 73 %; CR: 70 %; well tolerated
NCT03056339	I/II	MD Anderson	iC9/CAR.19/IL15	CD19	Cord blood	Relapsed/refractory CD19^+^ lymphomas	High response rates; no CRS/GvHD

CAR: chimeric antigen receptor; NK: natural killer; CR: complete response; ORR**:** overall response rate; GvHD: graft-versus-host disease; CRS: cytokine release syndrome; ICANS**:** immune effector cell-associated neurotoxicity syndrome; CD: cluster of differentiation; iNHL**:** indolent non-Hodgkin lymphoma; LBCL: large B-cell lymphoma; iPSC: induced pluripotent stem cell.

## AFM13 and natural killer cell combination therapy

Presently, ongoing research endeavors and clinical trials are dedicated to refining these aforementioned aspects of NK cell therapy with a primary focus on evaluating the safety, efficacy, and long-term outcomes in lymphoma patients. Affimed (AFMD), a clinical-stage immuno-oncology company, focuses on developing targeted immuno-oncology therapies. Affimed has three programs (AFM13, AFM24, and AFM26). AFM13 (acimtamig) is a first-in-class tetravalent bispecific innate cell engager, engineered as a tandem antibody (TandAb) construct to treat CD30-expressing malignancies. It facilitates targeted tumor lysis by providing bivalent binding to CD30 on malignant cells and CD16A on NK cells and macrophages. It recruits NK (NK) cells by binding to CD16A as immune effector cells [31]. AFM13 induces NK cell-mediated cytotoxicity, resulting in tumor cell lysis. Results of a Phase 1 dose-escalation study conducted on 28 patients with heavily pretreated relapsed or refractory HL show that AFM13 is well-tolerated and active, with an objective response rate of 50 %. Hence, AFM13 represents a new, feasible, targeted immunotherapy for heavily pretreated patients with HL. The dose regimen of AFM13 has to be optimized and the treatment duration has to be prolonged in order to increase the clinical efficacy [43]. The second study is an ongoing Phase II clinical trial that aims to evaluate the antitumor activity and safety of AFM13 in patients with CD30-positive peripheral T-cell lymphoma (PTCL) or transformed mycosis fungoides (tMF). The trial is investigating the efficacy of AFM13 as monotherapy in patients with relapsed or refractory CD30-positive T-cell lymphoma. The results of the trial are not yet available [44]. Peripheral T-cell lymphomas are highly aggressive and one of the most difficult-to-treat forms of lymphoma. Affimed, after underwhelming monotherapy data, has decided to focus on testing AFM13 in combination with the AB-101 NK cell product of Artiva. AB-101 NK is an allogeneic, non-genetically modified cord blood-derived NK cell. It is a cryopreserved NK cell therapy that has been optimized for combination with monoclonal antibodies to enhance ADCC and anti-tumor responses. The combination of AFM13 and AB-101 NK cells has the potential to provide a more durable and effective response compared to monotherapy with AFM13 [45].

## AFM13 and umbilical cord-derived natural killer cell combination

AFM13 is a tetravalent bispecific antibody engineered to engage simultaneously CD30 on cancer cells and CD16A on NK cells, thereby enhancing ADCC. To further improve its efficacy, more recent studies have focused on combining AFM13 with umbilical cord blood-derived NK cells, particularly those activated with cytokines to enhance their persistence and cytotoxic activity. Kerbauy et al. demonstrated in preclinical models that cytokine-induced memory-like NK cells derived from cord blood, which were precomplexed with AFM13, exhibited enhanced antitumor activity against CD30-positive lymphoma cells. The NK cells were hyperactivated and cytotoxic despite the presence of immunosuppressive cytokines such as TGF-β and IL-10, which are typically upregulated within the tumor microenvironment [46]. Thereafter, a Phase I/II first-in-human trial by Nieto et al. at MD Anderson Cancer Center evaluated the safety and efficacy of this combination in relapsed or refractory CD30-positive lymphomas. The trial consisted of *ex vivo* AFM13 precomplexing with cytokine-preactivated cord blood-derived NK cells before infusion. Preliminary outcomes report an ORR of 93 % and CR of 66 % with favorable safety outcomes [47]. These findings underscore the effectiveness of AFM13 combined with NK cells derived from umbilical cord blood as a potent method in the treatment of CD30-positive lymphomas, particularly in relapsed or refractory disease, and implicate the utility of innate immune effectors in next-generation cancer immunotherapies.

## Cytokine-induced memory-like natural killer cells in lymphoma therapy

One of the most important advances in NK cell-based immunotherapy is the generation of cytokine-induced memory-like NK (CIML-NK) cells. The cells are generated by a temporary pre-activation with IL-12, IL-15, and IL-18, which are capable of inducing memory-like functional properties. CIML-NK cells exhibit increased IFN-γ production, increased cytotoxicity, and increased proliferative capacity upon secondary stimulation. Phenotypically, they are CD56^bright^CD16^dim^ with strong expression of activating receptors (NKG2D, NKp30, and NKp46) and transcription factors (T-bet and Eomes) [48]. Epigenetic reprogramming following cytokine stimulation is a hallmark of CIML-NK cells, leading to lasting changes in phenotype and function [49]. The epigenetic modifications involve demethylation of regulatory elements in the IFNG locus (*e.g.*, CNS1) and regulation of master genes such as *PRDM1*/*BLIMP1* and *ZBTB32*, which together allow long-term IFN-γ expression in progeny cells as well as enhanced cytotoxic activity. This long-term reprogramming makes CIML-NK cells attractive candidates for cancer immunotherapy, particularly for treating refractory and relapsed lymphomas [50].

Despite their tremendous potential, there are currently few clinical trials evaluating CIML-NK cells in the context of lymphoma, and no specific studies have been formally published yet. Most clinical data come from a Phase I study (Clinical Trial: NCT01898793) that focuses primarily on patients with acute myeloid leukemia (AML) and myelodysplastic syndromes (MDS). In this study, NK cells were preactivated with IL-12, IL-15, and IL-18, and infused into patients to assess safety and preliminary efficacy. Although lymphoma was not the primary target, the study illustrated the broad potential of CIML-NK cells for the treatment of refractory hematological malignancies [43].

Preclinical research has further proved the efficacy of CIML-NK cells against lymphoma and melanoma models. In such experimental models, CIML-NK cells, which had been activated with IL-12, IL-15, and IL-18, were infused into mice with established tumors. The results demonstrated that CIML-NK cells effectively controlled tumor growth and increased survival rates, thus confirming their notable therapeutic value in treating tumor resistance to NK cells [51]. However, specialized clinical trials are needed to confirm these results in lymphoma patients.

Various combinatorial approaches have been established to further improve the activity of CIML-NK cell therapy. A combination of CIML-NK cells with monoclonal antibodies like rituximab (anti-CD20) has augmented ADCC and clinical responses considerably [52]. Moreover, strategic targeting of the immune checkpoint pathways suppressing NK cell function, specifically NKG2A and PD-1/PD-L1, is a novel approach in reconstituting CIML-NK cytotoxicity for the immunosuppressive tumor microenvironment [53]. Cytokine support strategies, specifically using IL-15 superagonists such as ALT-803 (*n* - 803), have been found to augment the *in vivo* stability, expansion, and activation of CIML-NK cells, resulting in durable antitumor activity [54].

Moreover, genetic modification of CIML-NK cells through the use of CARs has significantly enhanced their therapeutic efficacy. A recent preclinical study demonstrated that CIML-NK cells transduced with CD19-targeting CAR exhibited potent antitumor activity against CD19-expressing malignancies. In comparison to the conventional CAR-NK cells, CAR-CIML-NK cells produced more IFN-γ and exhibited greater degranulation against CD19^+^ target cells, resulting in greater cytotoxicity against leukemic and lymphomatous cells. Furthermore, the IL-12, IL-15, and IL-18 induced memory-like characteristics enhanced the *in vivo* persistence of CAR-CIML-NK cells and effectively inhibited tumor growth in CD19^+^ lymphoma mouse models, thereby resulting in extended survival [55]. For a succinct overview of the role of NK cells as a therapeutic intervention in lymphoma, pertinent studies and evidence are summarized in Table 2.

## Future trends

Various preclinical and clinical studies have demonstrated that improving the clinical efficacy of NK cell therapy for hematopoietic malignancies, such as lymphoma and leukemia, requires overcoming significant challenges related to optimal proliferation, long-term persistence, and effector function.

Appropriate selection of cell sources, advances in expansion methods and genetic modification techniques (CAR, Bispecific Killer Cell Engagers [BiKEs], high-affinity, non-cleavable CD16 [hnCD16]), cytokine adjuvants, antibody design and stem cell biology have been effective in overcoming these limitations.

Recently, induced pluripotent stem cells (iPSCs) and the NK-92 cell line have emerged as promising substrates for CAR engineering, owing to their inherent homogeneity and virtually unlimited proliferative capacity. However, due to the lymphoma origin of NK-92 cell lines, there are some limitations. Furthermore, technical complexities inherent to iPSC differentiation have hindered the large-scale manufacturing of NK cells from this progenitor source. However, regarding the potential and ability of NK 92 lines and iPSCs as an off-the-shelf therapy, advances in the use of these cells, expansion methods, and specific targeting employing genetic engineering have been made and are being investigated. These advances promise to overcome many challenges, thus bringing the application of NK 92 lines and iPSC-derived NK cells closer as clinically approved options for the treatment of hematologic and solid malignancies. Until now, the NK92 cell line has not been used in any registered trials, however, iPSC-derived NK cells have been used in Phase I clinical trials of lymphoma and other malignancies (Clinical Trials: NCT04245722; NCT04023071; NCT04630769; NCT0455188; NCT03841110). In recent years, engineered iPSC-derived NK cells have demonstrated promising results in preclinical lymphoma models. Specifically, FT516 was engineered to express a high-affinity CD16 receptor for targeting both CD19 and CD20 antigens. This platform was further optimized in FT596 as a triple-gene-engineered product incorporating these modifications alongside a membrane-bound 15/IL-15Rα fusion protein. Subsequent iterations have utilized transgenic overexpression of *ARID5B* or CRISPR-mediated knockout of *CD38* to enhance *in vivo* persistence and effector function.

Regarding expansion methods, it was observed that the *ex vivo* incubation of NK cells with IL-15 and nicotinamide (NAM) exhibited improved proliferation, persistence, cytotoxicity, and inflammatory cytokine production (*e.g.*, Gamida cell; GDA-201 - Clinical Trial: NCT05296525) [56]. In addition to cytokine-based approaches, feeder cell-based expansion has shown significant promise. For example, irradiated K562 feeder cells genetically engineered to express membrane-bound IL-15 (mbIL-15), IL-21 (mbIL-21), and 4–1BBL have been extensively used to promote robust NK cell expansion. These feeder-based protocols can achieve over 1000-fold expansion within two to three weeks and improve NK cell cytotoxicity and IFN-γ secretion. Feeder-supported methods have been used in good manufacturing practice-compliant protocols for clinical-grade NK cell production and are currently under evaluation in clinical trials (*e.g.*, NCT00995137, NCT02028407) [57]. Nevertheless, safety concerns regarding the tumor origin of feeder cell lines have led to parallel development of feeder-free systems and artificial antigen-presenting cells (aAPCs).

In recent years**,** CAR- and BiKE-NK cells have been used as alternative safer cells compared to CAR-T cells, for treating hematological malignancies. NK-92 cell lines represent one of the most widely utilized sources for CAR-NK cells; however, due to their limited *in vivo* expansion, umbilical cord blood (UCB) has emerged as a viable alternative. UCB-derived NK cells exhibit a higher proliferative rate and enhanced *in vivo* persistence compared to cell line-based models. Recently, several CAR-NK cell products have entered Phase I/II clinical trials for solid tumors and hematologic malignancies (Clinical Trials: NCT05654038, NCT05528341). Furthermore, promising Phase II trials are currently underway for patients with relapsed or refractory lymphoma who have previously failed brentuximab vedotin and PD-1 inhibitors. These studies, including LuminICE-203 (Clinical Trial: NCT05883449) and AFM13–104 (Clinical Trial: NCT04074746), utilize allogeneic cord blood-derived NK cells pre-complexed with AFM13, followed by multiple infusions of AFM13 monotherapy. These trials assess dose-escalation of pre-complexed, off-the-shelf NK cells, followed by expansion cohorts in Phase I/II for relapsed or refractory lymphoma, particularly CD30^+^ peripheral T-cell lymphoma.

Recent studies on the treatment of relapsed and refractory lymphoma suggest that, in addition to selecting optimal NK cell sources and enhancing *in vivo* persistence, combinatorial strategies, including the use of anti-lymphoma monoclonal antibodies, BiKEs, or checkpoint inhibitors, yield superior efficacy compared to monotherapeutic cell therapy. These improved outcomes are primarily mediated through more precise tumor targeting and the blockade of inhibitory regulators.

Preclinical trials using new NK products with improved expansion, demonstrate increased anti-lymphoma activity due to enhanced persistence and potency of transferred NK cells. The findings from these trials will impact the design of future clinical protocols; when integrated with multimodal strategies and the optimization of dosage and administration schedules, these advancements may lead to superior therapeutic outcomes in the treatment of lymphoma.

## Conclusion

NK cells are garnering significant attention in lymphoma therapy due to their unique properties, most notably their ability to exert rapid and potent antitumor responses without prior sensitization. However, the complex interplay between NK cells and the lymphoma microenvironment, coupled with immune escape mechanisms and the suppressive effects of certain medications, can impair NK cell function. Integrating NK cell therapy with chemotherapy or immune checkpoint inhibitors holds significant potential for synergistic effects and may overcome these hurdles. While limitations regarding optimal proliferation, long-term persistence, and effector function remain, recent advances are addressing these challenges. Specifically, the selection of appropriate cell sources, progress in expansion protocols, and genetic modifications, including CAR, BiKEs, and hnCD16, alongside cytokine adjuvants and stem cell biology, are proving effective in enhancing the therapeutic efficacy of NK cells.

## Funding

This project was supported by Hematopoietic Stem Cell Research Center, Shahid Beheshti University of Medical Sciences, Tehran, Iran.

## Data availability

The data that support the findings of this study are available from the corresponding author upon reasonable request.

## Conflicts of interest

We wish to confirm that there are no known conflicts of interest associated with this publication and there has been no significant financial support for this work that could have influenced its outcome
